# Feasibility Study of *Lactobacillus Plantarum 299v* Probiotic Supplementation in an Urban Academic Facility among Diverse Pregnant Individuals

**DOI:** 10.3390/nu15040875

**Published:** 2023-02-09

**Authors:** Nefertiti OjiNjideka Hemphill, Lacey Pezley, Alana Steffen, Gloria Elam, Michelle A. Kominiarek, Angela Odoms-Young, Nicollette Kessee, Alyshia Hamm, Lisa Tussing-Humphreys, Mary Dawn Koenig

**Affiliations:** 1 Department of Kinesiology and Nutrition, College of Applied Health Sciences, University of Illinois at Chicago, 1919 W. Taylor St., Chicago, IL 60612, USA; ojinjide@uic.edu (N.O.H.); lwissl2@uic.edu (L.P.); nkesse2@uic.edu (N.K.); ahamm6@uic.edu (A.H.); tussing@uic.edu (L.T.-H.); 2 Department of Population Health Nursing Science, College of Nursing, University of Illinois at Chicago, 845 S. Damen Ave, Chicago, IL 60612, USA; steffena@uic.edu; 3 Department of Obstetrics and Gynecology, College of Medicine, University of Illinois at Chicago, 820 S. Wood St., Chicago, IL 60612, USA; gelam@uic.edu; 4 Department of Obstetrics and Gynecology, Northwestern University Feinberg School of Medicine, 250 W. Superior St., Chicago, IL 60611, USA; mkominia@nm.org; 5 Division of Nutritional Sciences, College of Human Ecology, Cornell University, 116 Reservoir Ave, Ithaca, NY 14853, USA; odoms-young@cornell.edu; 6 Department of Human Development Nursing Science, College of Nursing, University of Illinois at Chicago, 845 S. Damen Ave, Chicago, IL 60612, USA

**Keywords:** iron, anemia, probiotic, *Lactobacillus plantarum* 299v, pregnancy

## Abstract

(1) Background: Despite iron intake recommendations, over a quarter of pregnant individuals have iron deficiency. *Lactobacillus plantarum 299v* (*LP299V*^®^) enhances iron absorption in non-pregnant populations and may have positive effects in pregnancy among those with sufficient iron stores; however, no studies have evaluated the effect of *LP299V*^®^ on maternal and neonatal iron status among individuals at risk for iron deficiency anemia in pregnancy. Thus, this study aims to assess the feasibility and preliminary efficacy of daily oral *LP299V*^®^ maternal supplementation among diverse pregnant individuals. (2) Methods: In this double-blind placebo-controlled randomized supplementation feasibility study, participants were randomized to probiotic *LP299V*^®^ + prenatal vitamin with iron or placebo + prenatal vitamin with iron from 15–20 weeks of gestation through delivery. (3) Results: Of the 20 enrolled and randomized participants, 58% (7/12) from the *LP299V*^®^ group and 75% (6/8) from the placebo group were retained. Adherence to supplementation was 72% for *LP299V*^®^/placebo and 73% for the prenatal vitamin. A slower decline in maternal hematological and iron parameters across pregnancy was observed in the *LP299V*^®^ group compared to placebo. (4) Conclusions: *LP299V*^®^ may be a tolerable therapy during pregnancy and has the potential to affect maternal and neonatal hematological and iron status.

## 1. Introduction

The most prevalent micronutrient deficiency in the United States (U.S.) is iron; a large majority of cases of iron deficiency (ID) and iron deficiency anemia (IDA) occur among pregnant women [1,2]. During pregnancy, maternal iron stores are used for the growing fetus, maternal red blood cell (RBC) expansion, and placental growth and development [3], thus increasing the risk for ID and IDA. Across all trimesters of pregnancy in the U.S., it is estimated that 18% of individuals have ID and 5% have IDA, and within the third trimester, the prevalence of ID exceeds 27%. Prevalence of IDA is even greater among those who identify as Black or low-income [2]. Maternal ID and IDA are associated with increased risk of preterm birth, low infant birth weight, maternal and fetal mortality, and irreversible infant neurocognitive defects [4,5]. To meet this increasing requirement for iron and to optimize maternal iron nutrition, the Recommended Dietary Allowance for pregnancy is 27 mg/day of iron [6]. However, given the continued high rates of maternal ID and IDA and only modest adherence to daily prenatal vitamins containing iron [7], alternative approaches to optimizing iron nutrition in pregnancy are needed.

Research has shown that one-time or short-term dosing of the probiotic *Lactobacillus plantarum 299v* (*LP299V*^®^) enhances iron absorption in non-pregnant populations [8,9,10,11]. However, few studies have examined the effect of long-term supplementation on body iron stores. While probiotics are considered safe to consume in pregnancy, only one *LP299V*^®^ supplementation trial has been conducted during the gestational period to evaluate its effects on maternal iron stores and risk of IDA [12]. This study, among iron-sufficient pregnant Swedish women, showed a significantly lower decline in iron stores and a significantly lower prevalence of IDA in the third trimester among those randomized to *LP299V*^®^ compared to standard care control [12]. 

These results offer potential positive effects for the role of *LP299V*^®^ in maintaining maternal iron status among those starting pregnancy with sufficient iron stores and who receive care in a decentralized publicly funded healthcare system [12]. However, no studies have evaluated the effect of *LP299V*^®^ on maternal iron status among individuals at risk for IDA in pregnancy in the U.S., nor have studies extended findings to neonatal iron status. Moreover, it is unknown if positive feasibility and preliminary efficacy would persist in a U.S.-based health care setting with racially, ethnically, and socioeconomically diverse pregnant individuals. Therefore, the objectives of this study were as follows. First and foremost, we examined the feasibility of daily oral *LP299V*^®^ maternal supplementation taken from the early second trimester through birth. Second, we explored the preliminary efficacy of *LP299V*^®^ intake on maternal (at-risk for IDA defined as hemoglobin (Hb) between 10.0–12.0 g/dL) and neonatal cord hematological and iron status parameters compared to controls in an urban U.S. academic medical center with a racially, ethnically, and socioeconomically diverse patient population.

## 2. Materials and Methods

Study design. This was a two-arm double-blind placebo-controlled randomized supplementation feasibility study conducted at an urban Midwest academic medical center. All participants provided written informed consent (IRB #2016-0662) prior to study participation. The study is registered at clinicaltrials.gov (NCT03646487). 

Research participants. Clinic schedules were reviewed daily to identify potentially eligible women. Initial eligibility was further assessed via electronic health records (EHR). Women identified as potentially eligible were approached in clinic or called to assess interest. Inclusion and exclusion criteria are presented in Table 1. 

Treatment groups. Women were randomly assigned to one of two treatment groups: probiotic *LP299V*^®^ + prenatal vitamin with iron (PNVI), or placebo + PNVI. All participants were instructed to consume the probiotic or placebo and PNVI daily from 15–20 weeks of gestation (WG) up through admittance for delivery. Participants were asked to consume supplements with a cool or room temperature beverage at the same time each day. Participants were able to choose what time of day to consume their supplements; however, it was suggested that the probiotic/placebo and the PNVI be taken together at bedtime or at the same time each day. We used NatureMade Digestive Probiotics, Daily Balance, containing hypromellous capsule material, potato starch, magnesium stearate, and 10^10^ CFUs *LP299V*^®^ sourced from ProbiAB, which has documented efficacy for enhancing iron absorption in non-pregnant reproductive-age women and for optimizing iron status among pregnant women [8,9,12]. The PNVI was from NatureMade and provided the % daily value for pregnant and lactating women in one daily tablet. Each PNVI tablet contained 27 mg of iron in the form of ferrous fumarate. The placebo was produced by the University of Illinois at Chicago (UIC) Investigational Drug Service (IDS). The placebo consisted of a 100% gelatin capsule containing cellulose Microcryst PH-102, a non-soluble fine white powder selected to resemble the *LP299V*^®^ supplement, while being non-absorbable. The placebo was indistinguishable from the probiotic to both participants and researchers. Both the probiotic/placebo capsules and PNVI were packaged in smart medication bottles (Pillsy, Seattle, WA, USA) and generically labeled as “Probiotic or Placebo” and “Prenatal Vitamin.” The medical director of Obstetrics and Gynecology provided a prescription for the supplements to be dispensed by UIC IDS. Women received approximately 32 pills at a time, or approximately one month’s supply. Refills occurred approximately every four weeks and were coupled with standard care clinic visits. In response to the COVID-19 pandemic, refills were shipped directly to the participant’s home. 

Randomization. Women were randomized at the baseline visit following a 2:1 allocation ratio using the Research Electronic Data Capture (REDCap^®^) randomization module. The biostatistician provided the randomization scheme to the UIC IDS, the party responsible for filling all supplement prescriptions, to ensure that the study recruitment and data collection staff remained blinded.

Data collection. Data was collected at baseline (15–20 WG), 24–28 WG, 34–36 WG, Labor and Delivery and during monthly pill refill visits that fell outside of the main study visits. Most data collection was conducted in person with modifications made at the onset of the COVID-19 pandemic as described below. 

Study feasibility. The electronic health record (EHR) was screened daily to identify eligible women scheduled for a “New Obstetrics” visit. Women were tracked, and the number of women approached (by phone & in person) for enrollment and the number of women who declined was documented. Once a woman was enrolled in the study, attendance at study visits, completeness of data, and overall and treatment specific loss to follow-up/withdrawal was closely monitored and documented. To track progress of participants through the study, the Consolidated Standards of Reporting Trials (CONSORT) subject flow diagram was utilized [13]. Study feasibility was defined *a priori* as recruitment ≥ 50% of those eligible, participants completing ≥80% of planned study visits, and retaining ≥80% of participants in both treatment arms through admittance for delivery. 

Health events. Health events including medication changes, iron supplementation, pregnancy-related conditions, and gastrointestinal symptoms were assessed monthly by survey and through viewing provider notes in the participant’s EHR. Study tolerability was defined *a priori* as no serious health events and the rate of non-serious health events being similar between study arms. 

Adherence monitoring. Adherence to the supplements was assessed using a Bluetooth-enabled smart pill bottle (Pillsy, Seattle, WA) and standard hand pill counts. At baseline, the smart pill bottles were paired with the participant’s smartphone by a member of the research staff, through the Pillsy phone application. Once paired, the application sent daily alerts to the subject when it was time to take the supplements (i.e., the pill bottle rang and lit up, and a secondary reminder was sent through the phone application). Data was also transmitted to a HIPAA-compliant research platform that tracked the number of times the bottle was opened with a date and timestamp. The technology allowed members of the research staff to receive daily dose-compliance information and allowed for two-way text and call communication between participants and study staff if data from the smart bottle were not transmitted. To proactively account for technological challenges related to the smart bottles, UIC IDS performed standard hand pill counts at each bottle return and recorded the number of pills taken and remaining in each bottle in a spreadsheet that was shared with research staff when the study was completed.

Maternal and Cord Blood Collection and Processing. At each data collection visit, maternal venous whole blood was obtained, with a portion processed for serum, to assess maternal iron, inflammatory and hematological parameters. At delivery, a venous umbilical cord blood sample was obtained at the bedside following delivery of the placenta. All maternal and cord blood samples were processed and stored at −80 °C or sent immediately to a commercial laboratory (Quest Diagnostics, Wood Dale, IL, USA) for analysis. 

Complete Blood Count (CBC). CBC with differential was measured in whole maternal or cord blood by electronic cell sizing/counting/cytometry/microscopy by a local commercial lab (Quest Diagnostics, Wood Dale, IL, USA). Hb, obtained from the CBC, was used to define trimester-specific maternal IDA, with a downward correction of 0.8 g/dL for Black women [6]. At the time of the study, it was recommended to use a race-adjusted cut-point for IDA. However, this race-adjusted cut-point was recently determined to be unfounded [14]. IDA ranges included ≤11 g/dL for the first trimester, ≤10.5 g/dL for the second trimester, and ≤11 g/dL for the third trimester. IDA ranges with the correction for Black women are ≤10.2 g/dL for the first trimester, ≤9.7 g/dL for the second trimester, and ≤10.2 g/dL for the third trimester [15]. Hb is the primary iron-related outcome for pilot trials, given that it is the most common clinical marker for iron/hematological status in pregnancy. 

Iron Status Parameters. Serum ferritin and iron were measured from maternal and cord serum by immunoassay and spectrophotometry by a local commercial lab (Quest Diagnostics, Wood Dale, IL, USA). Normal ranges for maternal ferritin are: 2nd trimester 2–230 ng/mL and 3rd trimester 0–116 ng/mL [16,17,18]. For serum iron, normal trimester-specific levels are: 1st trimester 72–143 μg/dL, 2nd trimester 44–178 μg/dL, and 3rd trimester 30–193 μg/dL [16]. For transferrin saturation, normal levels are: 1st not reported, 2nd trimester 10–44%, and 3rd trimester 5–37% [16]. 

C-reactive Protein. High-sensitivity CRP (hs-CRP), a common clinical marker of systemic inflammation, was measured via nephelometry from maternal and cord serum by a local commercial lab (Quest Diagnostics, Wood Dale, IL, USA).

Survey Interviews. Interview-administered surveys included a socio-demographics and health history questionnaire and a 24-h diet recall. The 24-h diet recall was conducted using the Nutrition Data System for Research (NDSR) software (University of Minnesota, Minneapolis, MN, USA) using a multiple-pass interview approach, to standardize collection of the data [19]. The consumption of dietary supplements was evaluated in concurrence with 24-h diet recall using the Dietary Supplement Assessment Module included in NDSR [20]. Data from the recall was used to quantify the average intake of dietary (from food) and supplemental iron across the gestational period. 

Maternal Anthropometrics. Maternal height was measured using a fixed stadiometer (baseline only) and weight at each gestational data collection visit using a calibrated digital scale (or via EHR during the COVID-19 pandemic) and at admittance for labor and delivery (from EHR). BMI was calculated as kg/m^2^. Pre-pregnancy weight was self-reported. 

Neonatal Characteristics. There is evidence that fetal sex, neonatal weight at delivery and gestational age at delivery can affect maternal and neonatal iron status and hematological parameters [21,22]. We obtained these variables from the participant’s EHR.

Data Management. Research staff entered all data directly into a REDCap (Vanderbilt University, Nashville, TN, USA) data structure. Standard checks for outliers, duplicates, and other errors associated with data entry were conducted. 

Power and Statistical Analysis. We aimed to recruit 24 individuals, with the assumption of 80% retention, resulting in a final recruitment sample of 20. With a 2:1 allocation ratio distribution between groups, our sample size is adequate to estimate parameters (e.g., standard deviation, mean change) needed to inform the design of a future efficacy trial. Confidence intervals for feasibility proportions such as retention and percent adherence have a maximum width of 0.27 if proportions are 0.5, and 0.22 with a proportion of 0.8 [23,24]. 

Statistical analysis was performed using Stata/BE (versions 15 and 17, College Station, TX, USA). Stata variables not normally distributed were transformed using natural log transformation, and data were presented as geometric means with confidence intervals. For data that could not be normalized with transformation, non-parametric tests were utilized. Given the feasibility nature of the study, the analysis was largely descriptive. For continuous variables, means, confidence intervals, medians, interquartile ranges, and standard deviations were reported. For categorical variables, frequencies and percentages were reported. The feasibility and supplement adherence data were reported by all randomized participants and by per protocol analysis (≥80% supplement adherence, completed the study). The maternal and cord blood hematological markers presented were from the per protocol analysis (≥80% supplement adherence, completed the study). For the maternal data, we visualized changes in the hematological and iron status parameters using “spaghetti” plots to provide a demonstration of marker changes over the gestational period. 

Differences between treatment groups (*LP299V*^®^ + standard PNVI versus placebo + standard PNVI) for continuous data were compared by *t*-test or the Wilcoxon rank-sum test for non-parametric data and via Fisher’s exact test for categorical data. Maternal iron-related parameters and inflammation at baseline, 24–28 WG, 34–36 WG and labor and delivery, were evaluated by *t*-tests. Infant iron status and hematological parameters at delivery were compared between mother and infant pairs via *t*-tests. Mean changes in scores for maternal iron-related parameters were calculated from baseline for each time point, 24–28 WG, 34–36 WG, and labor and delivery. All *p* values were based on a two-sided test of statistical significance accepted at the level of *p* < 0.05. Significance testing for within- and between-group differences in hematological and iron status markers was assessed; however, this feasibility study was not powered for these types of analyses. Therefore, our data were presented descriptively.

## 3. Results

### 3.1. Eligibility Screening and Recruitment

**Figure 1:**
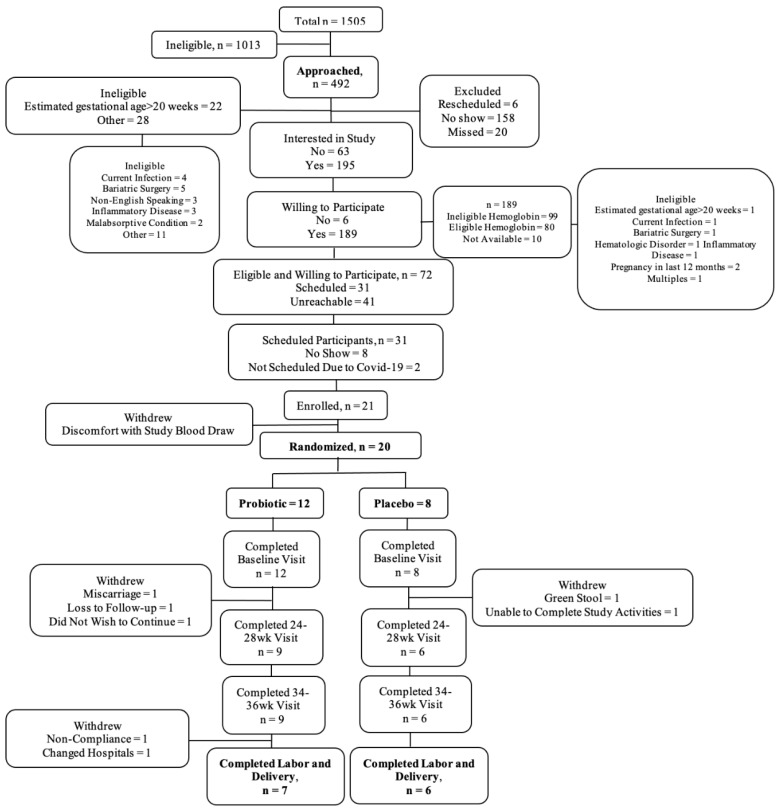
CONSORT flow diagram.

During the time of our study recruitment (January 2019–March 2020), a total of 1505 pregnant individuals scheduled their initial prenatal care visits at the University of Illinois Hospital and Health Sciences (UI Health) Center for Women’s Health. We screened all of these individuals’ electronic medical records to assess preliminary eligibility. Of those screened, 67% (1013/1505) were ineligible and the remaining 33% (492/1505) were preliminarily eligible and were approached. Of those willing to participate in the study (n = 195), 72 went on to meet full eligibility criteria (initial prenatal care visit Hb 10.0–12.0 g/dL). Of these, 21 individuals completed the informed consent process and were successfully enrolled into the study. Of those enrolled, 12 participants were randomized to the *LP299V*^®^ group and eight to the placebo group. Of the 20 enrolled and randomized participants, 58% (7/12) from the *LP299V*^®^ group and 75% (6/8) from the placebo group were retained. Completion of planned assessments for *LP299V*^®^ and placebo groups, respectively, were as follows: 100% (12/12) and 100% (8/8) at baseline, 75% (9/12) and 75% (6/8) at 24–28 WG, 58% (7/12) and 63% (5/8) at 34–36 WG, and 58% (7/12) and 75% (6/8) at delivery. Further details are provided in Figure 1. 

### 3.2. Participants

A total of 21 pregnant individuals were enrolled in the study. One participant withdrew prior to completing the baseline visit due to discomfort with study blood-draw activities, resulting in a total of 20 randomized participants. The mean maternal age was 28.9 years (SD 6.5) with a mean gestational age of 13.4 WG (SD 4.1) at baseline and 38.8 WG (SD 0.7) at delivery. The study population was comprised primarily of individuals who identified as non-Hispanic (16/20, 80%) or Black (15/20, 75%) women. Most individuals were either single and not living with a significant other (7/20, 35%) or married (7/20, 35%). More than half the participants had public health insurance (i.e., Medicaid) (11/20, 55%) and some high school or college education (15/20, 75%). Most participants reported a household income of less than or equal to $30,000 (14/20, 70%) and greater than half (11/20, 55%) were enrolled in the Supplemental Nutrition Assistance Program (SNAP). The mean pre-pregnancy BMI was 31.4 kg/m^2^ (SD 7.5) with over half the participants having pre-pregnancy obesity (11/20, 55%). At baseline, mean maternal BMI was 32.2 kg/m^2^ (SD 6.7) and indicative of a high degree of maternal obesity (12/20, 60%). A parity of 0 was reported by 40% (8/20) of participants with the remaining population divided between a parity of 1 (6/20, 30%) and 2 or more (6/20, 30%). The median intake of food iron was 10.5 mg per 1000 kcal (IQR 8.78) and total iron, food, and supplement together was 36.2 mg per 1000 kcal (IQR 22.1). The participants in the intervention groups differed for household income of ≤$30,000 (*p* = 0.005). All other baseline characteristics were similar (Table 2). 

### 3.3. Attrition

Following baseline, two (2/8) participants withdrew from the placebo group and five (5/12) from the probiotic group. Five participants withdrew during the 24–28 WG assessment period (three probiotic and two placebo) and two probiotic participants withdrew at delivery. The reasons for withdrawal differed for all seven participants and included bereavement/miscarriage, hospital change, did not wish to continue, green stool that was perceived to be linked to study supplement, unable to complete study activities; one participant was lost to follow-up and one withdrawn by the study staff for non-compliance. Baseline characteristics were largely similar between the completed and withdrawn groups, although dietary intake of iron was higher among the participants that withdrew from the study (data not shown). 

### 3.4. Supplement Adherence

Supplement adherence (Table 3) was measured using hand pill counts and pill bottle dosage monitoring software through Pillsy smart pill bottles. On average, participants took 72% of the *LP299V*^®^/placebo provided and 73% of the PNVI provided. Adherence was similar by randomization group. We also examined those who completed the study and observed no differences in adherence by treatment group. A difference was observed, however, for adherence by completed versus withdrawal status for *LP299V*^®^/placebo (*p* = 0.002) and PNVI (*p* = 0.003), with those remaining in the study being more adherent. 

### 3.5. General Adverse Events

Adverse events (AEs) were captured using the Maternal Adherence Form beginning with the first pill refill visit through delivery (Table 4). By randomization group, more upper respiratory conditions were observed in the placebo group compared to the *LP299V*^®^ group (*p* = 0.04). When stratified by withdrawal versus completed or completed by treatment group, reported AEs were similar. 

### 3.6. Adverse Pregnancy Conditions

At every research visit following baseline and excluding delivery, the use of antibiotics, iron supplements, the development of GDM, and the receipt of an intravenous iron infusion was monitored and recorded (Table 5). GI symptoms were also recorded using the GI Symptoms Checklist. The overall GI symptoms mean score was 42 (SD 24.2), and by randomization group, 41 (SD 29.2) for the *LP299V*^®^ group and 44 (SD 16.6) for the placebo group. When comparing participants by withdrawal group, those that completed the study had a higher (*p* = 0.04) mean GI symptoms score of 48 (SD 24.9), as compared to 24 (SD 7.8) for participants that withdrew.

### 3.7. Maternal Hematological and Iron Status Markers

We analyzed participants who completed the study with ≥80% adherence to examine changes in maternal hematological and iron status parameters by randomization group (Table 6). In this per protocol analysis, mean baseline concentrations of Hb, Hct, SI, TIBC, SF, and TSAT were the lowest and hs-CRP the highest among the *LP299V*^®^ group, a pattern that continued at all subsequent time points. 

Regarding the development of IDA, no participants in either treatment group displayed a Hb level indicative of IDA at 24–28 WG. However, at 34–36 WG, one participant in the *LP299V*^®^ group had IDA, and at delivery, one participant among the *LP299V*^®^ group and two participants in the placebo group had IDA. For this per protocol analysis, changes observed between treatment groups in absolute mean values and mean change values from baseline for all time points were similar. 

### 3.8. Neonatal Cord Hematological and Iron Status Markers

In the per protocol analysis, the neonatal hematological and iron status parameters were examined for the newborns of mothers with ≥80% adherence (Table 7). Gestational age at delivery, neonatal weight at delivery, and infant sex were not statistically different between treatment groups. We observed lower cord Hb, Hct, TIBC, similar SF, and higher SI and TSAT among neonates from the *LP299V*^®^ group. Cord hs-CRP concentrations were non-detectable.

## 4. Discussion

This study was a double-blind placebo-controlled feasibility RCT designed to examine the feasibility and preliminary efficacy of *LP299V*^®^ supplementation in pregnant individuals at-risk for IDA beginning ≤20 WG through delivery. Feasibility was the primary objective of this work given that, to our knowledge, this is the first study of its kind conducted in the U.S. and focused on pregnant individuals at risk for iron deficiency anemia and their neonates. 

*Recruitment and Enrollment.* Primary measures of feasibility were recruitment and enrollment, retention, and adherence to the intervention. Recruitment feasibility was defined as recruiting ≥50% of those eligible; however, only 15% (21/141) of fully eligible individuals were successfully enrolled into the study. Many (41/141, 29%) of these individuals were unreachable following their initial expression of interest and phone screening. While it is difficult to know why contact ceased, the OB patient population at UI Health largely identifies as low-income and minority individuals, and it is well documented that such circumstances involve barriers to participation in research including transportation, childcare, lack of time from other priorities of greater personal importance than study activities, and general distrust of the medical community [25,26,27]. Recruiting minority pregnant individuals can be especially challenging, as obstacles inherent to pregnancy (e.g., spousal approval and pregnancy-related problems) may be compounded by cultural and economic factors [28,29]. To proactively prepare for these potential issues, our team implemented recommended strategies [28,30], including race/ethnicity matching research staff to the target population when possible, using non-clinical language to explain the study, providing flexible scheduling based around prenatal visits, providing transportation for study visits as needed, offering limited child care with toys and activities for accompanying children, and providing financial incentives with the completion of study activities. 

We also worked with clinic staff and leadership to optimize recruitment and to integrate study activities into existing clinic processes. Many studies found the incorporation of primary care providers essential to the success of clinic-based perinatal research studies [28,31,32]. While clinic staff were willing to assist with in-facility logistics, our partnership fell short of true clinician buy-in, whereby procedures were not in place for clinical care providers to endorse the study or introduce the study staff member as a member of the health care team. This shortcoming likely contributed to the stunting of our recruitment numbers, as research has shown that people are more receptive to studies that are introduced by their provider [33]. One potential approach to elicit provider support for a future study would be to recruit clinicians interested in research during the development of the study, incorporate their feedback into the study design, and organize recruitment around their patient referrals. 

*Retention.* Our goal was to retain ≥80% of enrolled participants who would then complete ≥80% of planned assessments; however, only 58% (7/12) of participants from the *LP299V*^®^ group and 75% (6/8) from the placebo group were retained and no study visits had assessment completion rates of at least 80% for either group. Participants were diverse from an ethno-racial, education and economic perspective. Our findings showed a higher percentage of less educated and single women among those who were lost to follow-up or withdrawn from the study. This is similar to the 79% retention observed in Project DC-HOPE, a behavioral RCT designed to reduce smoking, depression, and intimate partner violence during pregnancy, which found that retention was lowest among women who were less educated and reported single relationship status [25]. The aforementioned study also recommended financial incentives, well-trained research staff, and consistent contact with participants as essential to longitudinal study retention [25]. All of these strategies were employed within our research protocol. Specific to our participants, we had difficulty scheduling and contacting two individuals with unstable housing, and we also had difficulty keeping engaged individuals with reported high family demands (e.g., caring for a child with disabilities and caring for multiple young children). Future studies might consider a screening period prior to randomization to gauge study engagement and commitment. This might include having the participants take a PNVI for a week to observe their compliance with the intervention. 

With transportation as another barrier and in response to the COVID-19 pandemic, we made several changes to study procedures: 1, remote study visits; 2, pill distribution via FedEx delivery; 3, pill adherence counts completed with study staff via video conference; 4, blood collection tubes were sent by mail and venous blood samples were collected during routine clinical phlebotomy visits, in place of the clinical research visit. These changes proved more convenient for participants and staff alike and appeared to ease participant study burden. 

*Adherence.* The a priori goal for adherence to the intervention was consumption of ≥80% of the supplements provided. Overall, adherence was 72% for the *LP299V*^®^/placebo and 73% for the PNVI. Among completers versus those that were withdrawn, adherence was 85% for the *LP299V*^®^/placebo and 86% for the PNVI. Similar adherence has been observed in other probiotic supplementation interventions conducted in pregnancy [34,35]. In a recent longitudinal RCT with 20 healthy Black and White pregnant individuals examining the feasibility of consuming the probiotic Florajen3 for prevention of group B *streptococcus* in pregnancy, adherence was 86% [36]. The authors used the electronic cap monitoring system MEMS to track subject supplement use, similar to the Pillsy electronic pill bottle system used in this study [36]. Unfortunately, half of the MEMS cap data was unusable, and two caps were not returned. Instead, the investigators had to rely on hand pill counts to calculate adherence, a less desirable method due to the potential for pill dumping before refill visits or unrecorded make-up doses consumed in response to missed doses [36]. Similar to their experience, we also had complications with the Pillsy technology. In our study, Pillsy smart caps were used in combination with pharmacy and participant hand pill counts (shifted to participant counts during the COVID-19 pandemic). While a small subset of participants experienced intermittent connectivity issues with Pillsy technology, the larger challenge was forgetting their assigned Pillsy bottles at pill refill visits. In this case, hand pill counts were used to rectify discrepancies in electronic data. Future studies should select a more portable bottle for participant convenience and provide a more in-depth Pillsy interface training at baseline to help eliminate usability issues. In the Swedish supplementation trial evaluating the effect of *LP299V*^®^ on iron status in healthy, iron-replete pregnant women, the authors reported 95% adherence among *LP299V*^®^ and 94% among placebo-treated groups [12]. However, it is unclear if the counts were subject to inconsistencies observed in other studies, as the authors did not report use of a secondary method to corroborate adherence results [12]. 

A more objective method, such as quantifying an increase in the probiotic strain in a fecal sample, should be considered. This approach was used in a probiotic supplementation study in pregnant Australian women with obesity to reduce the risk of GDM [37]. The measurement of probiotic in stool was used in conjunction with subject self-reporting of intake, and the results showed an adherence of 79% for the fecal samples and 90% for self-report [37]. This highlights the importance of a multi-method monitoring approach to accurately determine intervention adherence. 

*Adverse Events.* We hypothesized that AEs would be similar between the treatment arms. However, the placebo group had higher reports of upper respiratory infections (URIs). In pregnancy, hormonal changes often induce hyperemia, excess blood vessels in the sinus and nasal mucosa, and increased nasal cavity secretions, increasing risk of URIs [38]. Consistent with the only other *LP299V*^®^ supplementation trial in pregnancy [12], we observed no differences between groups for GI symptoms. However, when stratified by those who withdrew and completers, a higher GI symptom score was observed among study completers (*p* = 0.04). This was likely an artifact of increased GI discomfort associated with advanced gestation, given that completers reached full-term during the study as compared to participants that withdrew from the study earlier in the gestational period. 

*Maternal Iron Status Biomarkers.* Among participants included in the per protocol analysis, a slower decline in hematological and iron parameters across pregnancy was observed in the *LP299V*^®^ group compared to placebo. Although not powered to reliably detect small significant differences or associations, a positive pattern was observed in mean Hb changes from baseline across all study time points; baseline Hb levels were lower among the *LP299V*^®^ group but increased by delivery as compared to a decrease over time observed among the placebo group. 

Within the *LP299V*^®^ group, one participant presented with IDA at 34–36 WG and at delivery, while among the placebo group, there were no cases of IDA at 34–36 WG, and two participants presented with IDA at delivery. A similar observation was reported in the Axling study, where the prevalence of IDA was significantly lower among the *LP299V*^®^ group compared to placebo at 35 WG [12]. An important distinction to note is that the Axling study supplemented the gravida twice daily with *LP299V*^®^ and 12 mg ascorbic acid, 4.2 mg of iron and 30 µg folic acid [12]. Providing smaller boluses of vitamins and minerals provides a dietary absorption advantage over large one-time boluses. It is possible that supplementation of *LP299V*^®^ twice daily in conjunction with smaller amounts of iron and ascorbic acid (which facilitates iron absorption) could have potentially contributed to the positive findings observed among the pregnant women in the Axling trial [12]. Together, this suggests that regular consumption of *LP299V*^®^ may have positive effects on maternal iron nutrition and occurrence of IDA among those with iron-sufficiency or among those with subclinical ID early in pregnancy. However, a larger efficacy trial is required to confirm the positive pattern observed among the high-risk group targeted in our small feasibility study. 

Inflammation plays a pivotal role in iron metabolism, as its presence has the capacity to downregulate dietary uptake and initiate iron sequestration [39,40]. In the present study and the Axling trial, systemic inflammatory levels were captured using the acute-phase protein hs-CRP [12]. Participants in our study had higher hs-CRP levels compared to those in the Axling trial, likely due to the differences in BMI and participant sociodemographic characteristics between the two populations. The mean BMI in the Axling study was within normal range, while the participants in the current study had a high prevalence of obesity [12]. Higher levels of adiposity have been correlated with increased levels of inflammation and decreased iron bioavailability [41]. Moreover, our cohort was largely low-income Black women, who have been shown to have higher chronic psychosocial stressors in pregnancy compared with White women [42]. One proposed mechanism for this stress-related disparity is centered around the conceptualization of minority status as a chronic stressor [43]. Stress is directly correlated with inflammation [44,45] and likely more pervasive among our largely obese, minority population, as compared to that of the homogenous Swedish population. Although *LP299V*^®^ has been shown to have anti-inflammatory effects on the immune system [46], the possibility remains that elevated inflammatory levels can potentially complicate increased dietary iron absorption, presenting a greater challenge to the evaluation of *LP299V*^®^ efficacy in populations that are more inflamed. Given the beneficial effects of probiotic use in different clinical settings and patient populations with shared inflammatory and stress-related mechanisms, further exploration of the probiotic *LP299V*^®^ is warranted [47,48,49].

*Neonatal Iron Status Biomarkers.* Although not statistically significant, we observed higher SI and TSAT levels among neonates from the *LP299V*^®^ group compared to placebo. We observed similar SF concentrations between groups; however, all other biomarkers of iron (Hb, Hct, and TIBC) were higher among the placebo group. In pregnancy, neonatal iron needs increase with gestational age to establish fetal iron stores that are essential to ex utero neurodevelopment in the early months of life [3]. Fetal iron endowment relies exclusively on maternal iron transfer [50]. Previous research suggests that maternal-fetal iron trafficking is largely dictated by maternal signaling [51]. Moreover, a recent study among murine and in vitro human models indicates the placenta may also play a vital role in maternal-fetal iron transfer, responding to changes in maternal iron status [52]. These findings suggest that maintaining sufficient maternal iron status has important effects on placental functions and ultimately adequate iron transfer to the developing fetus [52]. As such, the results from our study are promising, given that the *LP299V*^®^ group began the study more iron-deficient than the placebo group yet improved in several hematological parameters over the course of pregnancy and delivered neonates with higher SI and TSAT levels than those in the placebo group. 

*Strengths and Limitations.* This study has several strengths. It is the first to assess the preliminary efficacy of *LP299V*^®^ in pregnant individuals with subclinical ID, a group at high risk for IDA, and among a racially, ethnically, socioeconomically diverse, U.S.-based population. It is also the first study to extend the evaluation of *LP299V*^®^ supplementation during pregnancy into neonatal hematological parameters and iron status at delivery. The study successfully followed participants from ≤20 WG through delivery with four data collection time points: baseline, 24–28 WG, 34–36 WG, and delivery. In addition, the collection of multiple iron and hematological parameters also helped to expand the examination of the preliminary effects of *LP299V*^®^ on iron nutrition in pregnancy. Lastly, the use of two-factor adherence monitoring using both the Pillsy smart bottle technology and pharmacy pill counts is a strength in regard to the accuracy of adherence measurement and subsequent reliability of the results.

There are several limitations of this study. This was a feasibility study in which the sample was too small to balance characteristics using randomization and to use multivariate methods to address imbalance. Additionally, due to the small sample size, this study was not powered to detect statistically significant changes in hematological and iron status parameters and limited our ability to conclusively determine study acceptability. Data collection challenges and protocol adaptions adopted in reaction to COVID-19 resulted in missing data. There were differences in Hb levels by group at baseline, a characteristic that we recommend be included in participant stratification for future projects. Although we excluded individuals using antibiotics in the past two months prior to enrollment, we did not withdraw participants if they started using antibiotics during the study, which could have affected the viability of *LP299V*^®^. Dietary data collected via self-reported 24-h recall is inherently subject to recall bias. Dose timing between meals should be considered to optimize iron absorption, and consumption with milk and other dairy products should be prohibited to limit chelation. Multi-day doses with smaller boluses of supplemental iron should be provided in place of one large daily bolus of iron from a PNVI. Additionally, key hematological markers of iron metabolism including hepcidin, sTfR, ERFE and EPO were not measured. Inclusion of sTfR would have provided unvarying insight into changes in plasma iron availability, as levels do not fluctuate in response to inflammation or pregnancy. Moreover, maternal-placental-fetal iron trafficking was not objectively measured and would have provided results on the direct effects of *LP299V*^®^ on this critical system.

## 5. Conclusions

The results of this study suggest the need for continued adjustments in the methods of recruitment and retention for probiotic supplementation among an urban U.S.-based health care setting. Once individuals were engaged in the research, there was strong adherence to the intervention and relatively few adverse events, indicating *LP299V*^®^ as a low cost and tolerable therapy during pregnancy. Preliminary findings suggest *LP299V*^®^ has the potential to affect several maternal and neonatal hematological and iron related parameters, and these findings should be further explored. 

## Figures and Tables

**Table 1:** Inclusion and exclusion criteria.

**Inclusion criteria:**
<20 weeks of gestation
18–45 years of age
At risk of maternal iron deficiency anemia defined as hemoglobin of 10.0–12.0 g/dL based on hemoglobin assessed at their initial prenatal care visit
Spontaneous/natural conception
Singleton pregnancy
Willing to refrain from all other supplements, including other prenatal vitamins with iron unless medically indicated (e.g., folate)
Refrain from other probiotic supplements (e.g., Activia, kefir)
Ability to read and write in English
Access to a smart phone
**Exclusion criteria:**
Autoimmune disorders
Current bacterial or viral infection
Oral antibiotic use in the past two months
Receiving steroid or anti-inflammatory treatment
Previous bariatric surgery
Malabsorptive condition
Current hyperemesis
Hematologic disorder (i.e., sickle cell disease or hemochromatosis)
High-dose iron supplementation
Current tobacco use
Current alcohol consumption
Current drug use
Type 1 or 2 diabetes

**Table 2:** Baseline demographic characteristics overall and by randomization group.

Variable	Overall (n = 20)	Probiotic (n = 12)	Placebo (n = 8)
Race, n (%) ^a^			
Black	15 (75)	10 (83)	5 (63)
White	5 (25)	2 (17)	3 (37)
Ethnicity, n (%) ^a^			
Non-Hispanic or Latino	16 (80)	11 (92)	5 (63)
Hispanic or Latino	4 (20)	1 (8)	3 (37)
Relationship status, n (%) ^a^			
Single not living with significant other	7 (35)	5 (42)	2 (25)
Single but living with significant other	6 (30)	3 (25)	3 (37)
Married	7 (35)	4 (33)	3 (37)
Health insurance, n (%) ^a^			
Private	7 (35)	6 (50)	1 (12)
Public	11 (55)	4 (33)	7 (88)
Other	2 (10)	2 (16)	0 (0)
Education, n (%) ^a^			
Some high school; some college	15 (75)	9 (75)	7 (88)
College graduate; graduate school	5 (25)	3 (25)	1 (12)
Household income, n (%) ^b^			
≤$30,999	14 (70)	7 (58)	7 (88)
≥$31,000	6 (30)	5 (42)	1 (12)
Receiving public assistance, n (%) ^a^			
WIC	5 (25)	3 (25)	2 (25)
SNAP	11 (55)	6 (50)	5 (63)
TANF	2 (10)	1 (8)	1 (12)
Maternal age, y ^b^	28.9 ± 6.5	29.0 ± 6.5	28.7 ± 6.9
Gestational age at baseline, WK ^b^	13 ± 4.1	12.6 ± 4.5	13.7 ± 3.7
Gestational age at delivery, WK ^b^	38.8 ± 0.7	38.6 ± 0.7	38.9 ± 0.7
Pre-pregnancy BMI (kg/m^2^) ^b^	31.4 ±7.5	31.1 ± 7.3	31.8 ± 8.3
Pre-pregnancy obesity, n (%) ^a^	11 (55)	7 (58)	4 (50)
Maternal BMI at baseline (kg/m^2^) ^b^	32.2 ± 6.7	31.7 ± 6.8	32.9 ± 6.8
Maternal obesity at baseline, n (%) ^a^	12 (60)	8 (67)	4 (50)
Parity, n (%) ^a^			
0	8 (40)	5 (42)	3 (37)
1	6 (30)	3 (25)	3 (37)
2	6 (30)	4 (33)	2 (25)
Food iron (mg/1000 kcal), median (IQR) ^b^	10.5 (8.7)	11.2 (4.3)	9.8 (12.9)
Total iron (food and supplement; mg/1000 kcal), median (IQR) ^b^	36.2 (22.1)	35.1 (23.9)	36.8 (16.7)

^a^ Fisher’s Exact Test. ^b^ Independent samples *t*-Test. BMI, Body Mass Index; WIC, Women Infants and Children Program; SNAP, Supplemental Nutrition Assistance Program; TANF, Temporary Assistance for Needy Families; IQR, Interquartile Range; WK, Week.

**Table 3:** Percent adherence for *LP299V*^®^/placebo and prenatal vitamin overall and by randomization, withdrawal, and completion groups.

	Overall, Mean (SD)	Randomization Group, Mean (SD)		Withdrawal Group, Mean (SD)		Completion Group, Mean (SD)	
% Adherence	Overall (n = 19)	Probiotic (n = 11)	Placebo (n = 8)	Withdrawn (n = 6)	Completed (n = 13)	Completed Probiotic (n = 7)	Completed Placebo (n = 6)
*LP299V*^®^/placebo	72 (27.5)	67 (29.1)	80 (25.3)	45 (22)	85 (19.4) *	79 (25)	93 (5.9)
Prenatal vitamin	73 (27.5)	67 (29.9)	81 (23.2)	45 (22.6)	86 (18.3) *	80 (23.6)	93 (5.1)

* The difference between withdrawn and completed groups for *LP299V*^®^/Placebo and Prenatal Vitamin were different, *p* = 0.002 and *p* = 0.003, respectively. The n = 19 reflects the withdrawal of one participant after baseline and prior to supplementation. SD, Standard deviation.

**Table 4:** General adverse events overall and by randomization, withdrawal, and completion groups.

		Randomization Group		Withdrawal Group		Completion Group	
Variable Category (*N*)	Overall (n = 19)	Probiotic (n = 11)	Placebo (n = 8)	Withdrawn (n = 6)	Completed (n = 13)	Completed Probiotic (n = 7)	Completed Placebo (n = 6)
Genitourinary conditions ^a^	13	8	5	1	12	7	5
Gastrointestinal symptoms	17	8	9	3	14	5	9
Upper respiratory conditions ^a,^*	6	1	5	0	6	1	5
Pain/swelling ^a^	15	8	7	2	13	6	7
Headaches/migraines ^a^	6	3	3	1	5	2	3
Problems sleeping ^a^	8	4	4	1	7	3	4
Emergency room visits ^a^	5	2	3	0	5	2	3
Acne/rash ^a^	2	1	1	1	1	0	1
Anxiety/depression ^a^	3	3	0	0	3	3	0
Fatigue/tired ^a^	7	5	2	2	5	3	2
Nosebleeds ^a^	3	0	3	0	3	0	3
Genetic iron diagnoses ^a^	2	1	1	0	2	1	1
Genetic fetal diagnoses ^a^	2	2	0	1	1	1	0
Other ^a^	4	2	2	2	2	0	2

(*N*) reports frequency of conditions per participant for each variable category. If more than one adverse event was experienced per category, the counts will exceed the participant (n) per group. Genitourinary Conditions include vaginal yeast infection (4), short cervix (1), trichomonas (3), continuous urinary tract infection (1), chlamydia (1), frequent urination (1), vaginitis (1) and ‘white stuff in urine’ (1). Gastrointestinal symptoms include abnormal oral glucose tolerance test (3), bloating (1), gassy (1), excessive stools (1), hemorrhoids (3), excessive saliva production (1), heart burn (1), nausea and food aversion (1). Upper Respiratory Conditions include shortness of breath (1), cold (1), asthma (2), sore throat (2). Pain/Swelling includes back pain (7), round ligament pain (2), leg pain (3), carpal tunnel (1) and swelling (2). Other includes ‘feeling warm’ (1), dizziness (2) and eyesight changes (1). All other categories include conditions as denoted by name. ^a^ Fisher’s Exact Test. * *p* = 0.04. The n = 19 reflects the withdrawal of one participant after baseline and prior to supplementation.

**Table 5:** Adverse pregnancy conditions overall and by randomization, withdrawal, and completion groups.

		Randomization Group		Withdrawal Group		Completion Group	
Variable Category (*N*)	Overall (n = 19)	Probiotic (n = 11)	Placebo (n = 8)	Withdrawn (n = 6)	Completed (n = 13)	Completed Probiotic (n = 7)	Completed Placebo (n = 6)
Blood transfusion	1	0	1	0	1	0	1
Antibiotic use ^a^	8	5	3	2	6	3	3
Iron supplementation ^a^	6	4	2	2	4	2	2
Gastrointestinal Symptoms Score, mean (SD) ^b^	42 ± 24.2	41 ± 29.2	44 ± 16.6	24 ± 7.8	48 ± 24.9 *	49 ± 31.7	46 ± 16.8
Gestational diabetes mellitus ^a^	3	3	0	1	2	2	0

^a^ Fisher’s Exact Test. ^b^ Independent samples *t*-Test. * *p* = 0.04. The n = 19 reflects the withdrawal of one participant after baseline and prior to supplementation. SD, standard deviation.

**Table 6:** Iron status biomarkers for completers with ≥80% adherence by randomization group at each time point and mean change from baseline to 24–28 weeks, 34–36 weeks, and labor & delivery.

	Probiotic			Placebo		
Biomarkers by Time Point	n	Absolute Value, Mean (95% CI)	Change from Baseline, Mean (95% CI) ^c^	n	Absolute Value, Mean (95% CI)	Change from Baseline, Mean (95% CI) ^c^
Hemoglobin (g/dL) ^a,d^						
Baseline	4	10.9 (10.1–11.7)		6	11.3 (10.6–12)	
24–28 WG	5	11.0 (10.1–11.9)	−0.03 (−0.68–0.63)	6	11 (10.6–11.5)	−0.2 (−0.8–0.4)
34–36 WG	4	10.9 (10.4–11.3)	0.1 (−0.2–0.4)	5	11.3 (10.3–12.4)	0.1 (−0.4–0.6)
L&D	5	11.2 (10.0–12.4)	0.4 (−1.1–1.8)	4	10.9 (9.0–12.7)	−0.1 (−1.7–1.6)
Hematocrit (%) ^a,d^						
Baseline	3	32.8 (29.1–36.5)		6	34.2 (31–37.4)	
24–28 WG	5	32.6 (29.9–35.3)	−1.2 (−3.5–1.1)	6	33.1 (31.7–34.5)	−1.1 (−3.4–1.2)
34–36 WG	4	32.3 (30.0–34.7)	−1.0 (−4.9–3.0)	5	34.5 (30.2–38.7)	0.5 (−1.4–2.3)
L&D	5	33.1 (30.2–35.9)	−0.03 (−6.6–6.5)	4	32.2 (26.8–37.7)	−0.5 (−4.7–3.8)
Serum iron (µg/dL) ^b,d^						
Baseline	5	73.5 (43.1–125.2)		6	100.1 (59.4–168.5)	
24–28 WG	5	89.3 (61.0–130.7)	13.4 (−38.3–65.1)	6	88.1 (51.1–151.9)	−13.7 (−57–29.7)
34–36 WG	4	83.2 (36.8–187.9)	14.0 (−25.6–53.6)	5	66.3 (33.7–130.5)	−11.6 (−36.5–13.3)
L&D	5	69.6 (42.8–113.4)	−5.2 (−17.6–7.2)	3	93.1 (57.0–153.0)	5.7 (−48.8–60.2)
Total iron binding capacity (µmol/L) ^a,d^						
Baseline	5	354.0 (308.6–399.4)		6	390.8 (352.8–428.9)	
24–28 WG	5	405.4 (354.4–456.4)	51.4 (−15.1–117.9)	6	441.8 (399.6–484.1)	51.0 (11.5–90.5)
34–36 WG	4	450.0 (364.9–535.1)	100.5 (10.7–190.3)	5	483.6 (439.4–527.8)	100 (53.2–147.60
L&D	5	443.4 (379.6–507.2)	89.4 (12.4–166.4)	3	461.0 (405.0–517.0)	58.3 (11.4–105.2)
Serum ferritin (ng/mL) ^b,d^						
Baseline	5	17.7 (9.9–31.8)		6	25.9 (13.2–51.0)	
24–28 WG	5	12.4 (7.3- 20.8)	−6.0 (−15.2–3.2)	6	18.9 (15.6–22.9)	−12.0 (−34.7–10.7)
34–36 WG	4	11.8 (6.9–20.1)	−5.8 (−14.3–2.8)	5	15.3 (7.8–30.0)	−17.2 (−49.4–15.0)
L&D	5	13.8 (8.3–23.2)	−4.4 (−9.4–0.6)	3	30.8 (10.4–91.5)	0 (−94.4–94.4)
Transferrin saturation (%) ^b,d^						
Baseline	5	20.9 (12.1–36.1)		6	25.8 (15.7–42.2)	
24–28 WG	5	22.0 (13.9–34.8)	0.6 (−16.4–17.6)	6	20.0 (11.6–34.4)	−6.0 (−15.3–3.3)
34–36 WG	4	18.9 (10.1–35.3)	−2.5 (−14.5–9.5)	5	13.4 (6.1–29.4)	−7.2 (−12.0–−2.4)
L&D	5	15.6 (9.9–24.6)	−6.2 (−15.1–2.7)	3	20.4 (12.0– 34.61	−1.7 (−12.0–8.7)
hs-CRP (mg/L) ^b,d^						
Baseline	5	5.8 (2.6–12.5)		6	4.8 (1.8–12.8)	
24–28 WG	5	5.4 (1.5–18.9)	0.4 (−2.5–3.3)	6	5.3 (2.1–13.5)	0.2 (−2.3–2.8)
34–36 WG	4	5.3 (3.0–9.3)	−0.8 (−4.1–2.4)	5	3.7 (0.9–15.6)	−1.6 (−5.7–2.5)
L&D	5	6.3 (4.2–9.5)	−0.04 (−3.9- 3.9)	3	4.6 (2.4–9.0)	−3.0 (−14.1–8.1)
IDA (n)						
Baseline	5	0		6	0	
24–28 WG	5	0		6	0	
34–36 WG	4	1		5	0	
L&D	5	1		4	2	

^a^ Arithmetic mean absolute values are presented. ^b^ Geometric mean absolute values are presented. ^c^ Estimated mean change in the difference between the baseline and follow-up means. ^d^ Independent *t*-test. Sample size (n) varies by time point and reflects available data for the corresponding study visit. CI, confidence interval; hs-CRP, high-sensitivity C-reactive protein; L&D, labor and delivery; WG, weeks of gestation; IDA, Iron Deficiency Anemia.

**Table 7:** Neonatal demographic characteristics and hematological and iron status biomarkers for ≥80% adherence by treatment group.

	Probiotic		Placebo	
Biomarkers	n	Absolute Value, Mean (95% CI)	n	Absolute Value, Mean (95% CI)
Gestational age at delivery (WG) ^a,c^	5	38.5 (37.6–39.4)	5	39.1 (38.2–40.0)
Weight at delivery (kg) ^a,c^	5	3.5 (3.3–3.6)	5	3.2 (2.8–3.5)
Neonatal sex ^d^ (n, male)	5	2	5	3
Hemoglobin (g/dL) ^a,c^	5	14.7 (12.1–17.3)	4	16.4 (14.6–18.2)
Hematocrit (%) ^a,c^	5	44.3 (38.5–50.2)	4	49.5 (44.9–54.0)
Serum iron (µg/dL) ^a,c^	5	136.2 (93.4–179.0)	3	108.0 (48.4–167.6)
Total iron binding capacity (µmol/L) ^a,c^	5	223.6 (155.6–291.6)	3	274.0 (97.3–450.7)
Serum ferritin (ng/mL) ^a,c^	5	88.8 (7–170.6)	3	88.0 (44.1–132.0)
Transferrin saturation (%) ^a,c^	5	63.8 (35.3–92.3)	3	43.3 (−12.6–99.2)
hs_CRP(mg/L) ^a,b^	5	ND	3	ND

^a^ Arithmetic mean values are presented. ^b^ hs_CRP levels were non-detectable (ND). ^c^ Independent samples *t*-test. ^d^ Fisher’s exact test. CI, confidence interval; hs-CRP, high-sensitivity C-reactive protein; WG, weeks of gestation. Sample size (n) varies by biomarker and reflects available data for the corresponding study visit.

## Data Availability

The data presented in this study are available on request from the corresponding author. The data are not publicly available due to participant privacy.

## References

[1] Gupta P.M., Hamner H.C., Suchdev P.S., Flores-Ayala R., Mei Z. (2017). Iron status of toddlers, nonpregnant females, and pregnant females in the United States. Am. J. Clin. Nutr..

[2] Adebisi O.Y., Strayhorn G. (2005). Anemia in pregnancy and race in the United States: Blacks at risk. Fam. Med..

[3] Bothwell T.H. (2000). Iron requirements in pregnancy and strategies to meet them. Am. J. Clin. Nutr..

[4] Lozoff B., Beard J., Connor J., Felt B., Georgieff M., Schallert T. (2006). Long-Lasting Neural and Behavioral Effects of Iron Deficiency in Infancy. Nutr. Rev..

[5] Allen L.H. (2000). Anemia and iron deficiency: Effects on pregnancy outcome. Am. J. Clin. Nutr..

[6] Institute of Medicine (2001). Dietary Reference Intakes for Vitamin A, Vitamin K, Arsenic, Boron, Chromium, Copper, Iodine, Iron, Manganese, Molybdenum, Nickel, Silicon, Vanadium, and Zinc.

[7] Cogswell M.E., Parvanta I., Ickes L., Yip R., Brittenham G.M. (2003). Iron supplementation during pregnancy, anemia, and birth weight: A randomized controlled trial. Am. J. Clin. Nutr..

[8] Hoppe M., Önning G., Hulthén L. (2017). Freeze-dried Lactobacillus plantarum 299v increases iron absorption in young females—Double isotope sequential single-blind studies in menstruating women. PLoS ONE.

[9] Hoppe M., Önning G., Berggren A., Hulthén L. (2015). Probiotic strain Lactobacillus plantarum 299v increases iron absorption from an iron-supplemented fruit drink: A double-isotope cross-over single-blind study in women of reproductive age. Br. J. Nutr..

[10] Bering S., Suchdev S., Sjoltav L., Berggren A., Tetens I., Bukhave K. (2006). A lactic acid-fermented oat gruel increases non-haem iron absorption from a phytate-rich meal in healthy women of childbearing age. Br. J. Nutr..

[11] Scheers N., Rossander-Hulthen L., Torsdottir I., Sandberg A.S. (2016). Increased iron bioavailability from lactic-fermented vegetables is likely an effect of promoting the formation of ferric iron (Fe^3+^). Eur. J. Nutr..

[12] Axling U., Önning G., Combs M.A., Bogale A., Högström M., Svensson M. (2020). The Effect of Lactobacillus plantarum 299v on Iron Status and Physical Performance in Female Iron-Deficient Athletes: A Randomized Controlled Trial. Nutrients.

[13] Rennie D. (2001). CONSORT Revised—Improving the Reporting of Randomized Trials. JAMA.

[14] Hamm R.F., Wang E.Y., Levine L.D., Srinivas S.K. (2021). Association Between Race and Hemoglobin at Delivery or Need for Transfusion When Using Race-Based Definitions for Treatment of Antepartum Anemia. Obstet. Gynecol..

[15] Committee on the Prevention and Management of Iron Deficiency Anemia among U.S. Children and Women of Childbearing Age (1994). Iron Deficiency Anemia: Recommended Guidelines for the Prevention, Detection, and Management among U.S. Children and Women of Childbearing Age.

[16] Abbassi-Ghanavati M., Greer L.G., Cunningham F.G. (2009). Pregnancy and laboratory studies: A reference table for clinicians. Obstet. Gynecol..

[17] World Health Organization (2007). Assessing the Iron Status of Populations: Including Literature Reviews. World Health Organization.

[18] Næss-Andresen M.-L., Eggemoen Å.R., Berg J.P., Falk R.S., Jenum A.K. (2019). Serum ferritin, soluble transferrin receptor, and total body iron for the detection of iron deficiency in early pregnancy: A multiethnic population-based study with low use of iron supplements. Am. J. Clin. Nutr..

[19] Dennis B., Ernst N., Hjortland M., Tillotson J., Grambsch V. (1980). The NHLBI nutrition data system. J. Am. Diet. Assoc..

[20] Harnack L., Stevens M., Van Heel N., Schakel S., Dwyer J.T., Himes J. (2008). A computer-based approach for assessing dietary supplement use in conjunction with dietary recalls. J. Food Compos. Anal..

[21] Phillips A.K., Roy S.C., Lundberg R., Guilbert T.W., Auger A.P., Blohowiak S.E., Coe C.L., Kling P.J. (2014). Neonatal iron status is impaired by maternal obesity and excessive weight gain during pregnancy. J. Perinatol..

[22] Viteri F.E. (2011). Iron endowment at birth: Maternal iron status and other influences. Nutr. Rev..

[23] Newcombe R.G. (1998). Two-sided confidence intervals for the single proportion: Comparison of seven methods. Stat. Med..

[24] Fleiss J.L. (2003). Statistical Methods for Rates and Proportions.

[25] El-Khorazaty M.N., Johnson A.A., Kiely M., El-Mohandes A.A., Subramanian S., Laryea H.A., Murray K.B., Thornberry J.S., Joseph J.G. (2007). Recruitment and retention of low-income minority women in a behavioral intervention to reduce smoking, depression, and intimate partner violence during pregnancy. BMC Public Health.

[26] Shavers-Hornaday V.L., Lynch C.F., Burmeister L.F., Torner J.C. (1997). Why are African Americans under-represented in medical research studies? Impediments to participation. Ethn. Health.

[27] Shavers V.L., Lynch C.F., Burmeister L.F. (2002). Racial differences in factors that influence the willingness to participate in medical research studies. Ann. Epidemiol..

[28] Goff S.L., Youssef Y., Pekow P.S., White K.O., Guhn-Knight H., Lagu T., Mazor K.M., Lindenauer P.K. (2016). Successful strategies for practice-based recruitment of racial and ethnic minority pregnant women in a randomized controlled trial: The IDEAS for a healthy baby study. J. Racial Ethn. Health Disparities.

[29] Gamble A., Beech B.M., Blackshear C., Cranston K.L., Herring S.J., Moore J.B., Welsch M.A. (2021). Recruitment planning for clinical trials with a vulnerable perinatal adolescent population using the Clinical Trials Transformative Initiative framework and principles of partner and community engagement. Contemp. Clin. Trials.

[30] Coleman-Phox K., Laraia B.A., Adler N., Vieten C., Thomas M., Epel E. (2013). Recruitment and retention of pregnant women for a behavioral intervention: Lessons from the maternal adiposity, metabolism, and stress (MAMAS) study. Prev. Chronic Dis..

[31] Frew P.M., Saint-Victor D.S., Isaacs M.B., Kim S., Swamy G.K., Sheffield J.S., Edwards K.M., Villafana T., Kamagate O., Ault K. (2014). Recruitment and retention of pregnant women into clinical research trials: An overview of challenges, facilitators, and best practices. Clin. Infect. Dis..

[32] Kerver J.M., Elliott M.R., Norman G.S., Sokol R.J., Keating D.P., Copeland G.E., Johnson C.C., Cislo K.K., Alcser K.H., Kruger-Ndiaye S.R. (2013). Pregnancy recruitment for population research: The national children’s study vanguard experience in Wayne County, Michigan. Paediatr. Perinat. Epidemiol..

[33] Newington L., Metcalfe A. (2014). Factors influencing recruitment to research: Qualitative study of the experiences and perceptions of research teams. BMC Med. Res. Methodol..

[34] Davidson S.J., Barrett H.L., Price S.A., Callaway L.K., Dekker Nitert M. (2021). Probiotics for preventing gestational diabetes. Cochrane Database Syst. Rev..

[35] Lindsay K.L., Brennan L., McAuliffe F.M. (2014). Acceptability of and compliance with a probiotic capsule intervention in pregnancy. Int. J. Gynecol. Obstet..

[36] Hanson L., Vandevusse L., Duster M., Warrack S., Safdar N. (2014). Feasibility of oral prenatal probiotics against maternal group B streptococcus vaginal and rectal colonization. JOGNN—J. Obstet. Gynecol. Neonatal Nurs..

[37] Callaway L.K., McIntyre H.D., Barrett H.L., Foxcroft K., Tremellen A., Lingwood B.E., Tobin J.M., Wilkinson S., Kothari A., Morrison M. (2019). Probiotics for the prevention of gestational diabetes mellitus in overweight and obese women: Findings from the SPRING double-blind randomized controlled trial. Diabetes Care.

[38] Rosenberger K.D., Cibulka N.J., Barron M.L. (2022). Guidelines for Nurse Practitioners in Ambulatory Obstetric Settings.

[39] Ganz T., Nemeth E. (2015). Iron homeostasis in host defence and inflammation. Nat. Rev. Immunol..

[40] Papanikolaou G., Pantopoulos K. (2017). Systemic iron homeostasis and erythropoiesis. IUBMB Life.

[41] Flores-Quijano M.E., Vega-Sánchez R., Tolentino-Dolores M.C., López-Alarcón M.G., Flores-Urrutia M.C., López-Olvera A.D., Talavera J.O. (2019). Obesity Is Associated with Changes in Iron Nutrition Status and Its Homeostatic Regulation in Pregnancy. Nutrients.

[42] Gillespie S.L., Porter K., Christian L.M. (2016). Adaptation of the inflammatory immune response across pregnancy and postpartum in Black and White women. J. Reprod. Immunol..

[43] Christian L.M., Glaser R., Porter K., Iams J.D. (2013). Stress-induced inflammatory responses in women: Effects of race and pregnancy. Psychosom. Med..

[44] Slavich G.M., Irwin M.R. (2014). From stress to inflammation and major depressive disorder: A social signal transduction theory of depression. Psychol. Bull..

[45] Redpath N., Rackers H.S., Kimmel M.C. (2019). The Relationship Between Perinatal Mental Health and Stress: A Review of the Microbiome. Curr. Psychiatry Rep..

[46] Wells J. (2011). Immunomodulatory mechanisms of lactobacilli. Microb. Cell Factories.

[47] Castelli V., D’Angelo M., Quintiliani M., Benedetti E., Cifone M.G., Cimini A. (2021). The emerging role of probiotics in neurodegenerative diseases: New hope for Parkinson’s disease?. Neural Regen. Res..

[48] Wu H., Chiou J. (2021). Potential benefits of probiotics and prebiotics for coronary heart disease and stroke. Nutrients.

[49] Vivarelli S., Falzone L., Basile M., Nicolosi D., Genovese C., Libra M., Salmeri M. (2019). Benefits of using probiotics as adjuvants in anticancer therapy (Review). World Acad. Sci. J..

[50] Cao C., Fleming M.D. (2016). The placenta: The forgotten essential organ of iron transport. Nutr. Rev..

[51] Dao M.C., Sen S., Iyer C., Klebenov D., Meydani S.N. (2013). Obesity during pregnancy and fetal iron status: Is Hepcidin the link?. J. Perinatol..

[52] Sangkhae V., Fisher A.L., Wong S., Koenig M.D., Tussing-Humphreys L., Chu A., Lelić M., Ganz T., Nemeth E. (2020). Effects of maternal iron status on placental and fetal iron homeostasis. J. Clin. Investig..

