# Correction: OjiNjideka Hemphill et al. Feasibility Study of *Lactobacillus Plantarum 299v* Probiotic Supplementation in an Urban Academic Facility among Diverse Pregnant Individuals. *Nutrients* 2023, *15*, 875

**DOI:** 10.3390/nu15153339

**Published:** 2023-07-27

**Authors:** Nefertiti OjiNjideka Hemphill, Lacey Pezley, Alana Steffen, Gloria Elam, Michelle A. Kominiarek, Angela Odoms-Young, Nicollette Kessee, Alyshia Hamm, Lisa Tussing-Humphreys, Mary Dawn Koenig

**Affiliations:** 1 Department of Kinesiology and Nutrition, College of Applied Health Sciences, University of Illinois at Chicago, 1919 W. Taylor St., Chicago, IL 60612, USA; 2 Department of Population Health Nursing Science, College of Nursing, University of Illinois at Chicago, 845 S. Damen Ave, Chicago, IL 60612, USA; 3 Department of Obstetrics and Gynecology, College of Medicine, University of Illinois at Chicago, 820 S. Wood St., Chicago, IL 60612, USA; 4 Department of Obstetrics and Gynecology, Northwestern University Feinberg School of Medicine, 250 W. Superior St., Chicago, IL 60611, USA; 5 Division of Nutritional Sciences, College of Human Ecology, Cornell University, 116 Reservoir Ave, Ithaca, NY 14853, USA; 6 Department of Human Development Nursing Science, College of Nursing, University of Illinois at Chicago, 845 S. Damen Ave, Chicago, IL 60612, USA

## Text Correction

There was an error in the original publication. LP299V^®^ is a registered trademark by Probi AB and this was not indicated in the original publication [1]. A correction has been made to all LP299V^®^ references throughout the text and tables: “*Lp299v*” should be replaced with “*LP299V*^®^”.

The authors apologize for any inconvenience caused and state that the scientific conclusions are unaffected. This correction was approved by the Academic Editor. The original publication has also been updated.
